# Health systems around the world – a comparison of existing health system rankings

**DOI:** 10.7189/jogh.08.010407

**Published:** 2018-06

**Authors:** Stefanie Schütte, Paula N Marin Acevedo, Antoine Flahault

**Affiliations:** 1 Centre Virchow-Villermé, Université Sorbonne Paris Cité, Charité-Universitätsmedizin Berlin, France-Germany; 2 Institute of Global Health, Faculty of Medicine, University of Geneva, Geneva, Switzerland

## Abstract

**Background:**

Existing health systems all over the world are different due to the different combinations of components that can be considered for their establishment. The ranking of health systems has been a focal points for many years especially the issue of performance. In 2000 the World Health Organization (WHO) performed a ranking to compare the Performance of the health system of the member countries. Since then other health system rankings have been performed and it became an issue of public discussion. A point of contention regarding these rankings is the methodology employed by each of them, since no gold standard exists. Therefore, this review focuses on evaluating the methodologies of each existing health system performance ranking to assess their reproducibility and transparency.

**Methods:**

A search was conducted to identify existing health system rankings, and a questionnaire was developed for the comparison of the methodologies based on the following indicators: (1) General information, (2) Statistical methods, (3) Data (4) Indicators. Overall nine rankings were identified whereas six of them focused rather on the measurement of population health without any financial component and were therefore excluded. Finally, three health system rankings were selected for this review: “Health Systems: Improving Performance” by the WHO, “Mirror, Mirror on the wall: How the Performance of the US Health Care System Compares Internationally” by the Commonwealth Fund and “the Most efficient Health Care” by Bloomberg.

**Results:**

After the completion of the comparison of the rankings by giving them scores according to the indicators, the ranking performed the WHO was considered the most complete regarding the ability of reproducibility and transparency of the methodology.

**Conclusions:**

This review and comparison could help in establishing consensus in the field of health system research. This may also help giving recommendations for future health rankings and evaluating the current gap in the literature.

Identifying simple, practical and understandable ways to assess health system performance, with its complex interlinked dimensions, remains a challenging goal. Health systems are complex, may be seen as the sum of all the organizations, institutions and resources whose primary purpose is to improve health with limited resources [1,2].

All health systems are different due to the different combinations of components they can consider. Ranking health systems is important for informing policy-makers and for strengthening health systems as well as prompt attention to inequalities amongst different populations. It is also in the interest of the United Nations (UN) and the World Health Organization (WHO) for systems to be assessed and compared for policies to be developed and so that the Sustainable Development Goals signed by the 193 member countries can be achieved [3]. Efficiency of a health system is often considered as the degree of achievement of the goals of a health system given the resources utilized to achieve these goals [4].

More than a decade ago, the WHO was pioneer in conducting the first health system performance ranking of the 191 member nations of the WHO [5]. They focused on how nations could improve the efficiency of health system performance by development of evidence based on the outcomes of health systems and their determinants [6]. This served as the basis for many rankings focusing on the performance of health systems. The methods for this ranking were published in a series of discussion papers by the WHO [7-10].

However, the performance of rankings may be a very complex process. First, a set of appropriate and available indicators such as health-relevant measures to represent the inputs and outputs of the systems has to be identified. Second, different weights, usually based on surveys, on statistical methods or on a collective decision among experts [11,12] are assigned to each indicator. Finally, statistical analyses are conducted to obtain the scores of health systems.

There exists several rankings for health systems and the main difference amongst them is the methodology used to conduct the ranking. As far as we know neither methodological gold standard nor consensus for the methodology to be used to conduct a ranking for health systems does exist. Indeed, while rankings are a popular method for comparison, there is much confusion and debate over which indicators to use and how to present the information in ranked format. Moreover, transparency is essential to the success of any ranking system. The openness of the process in terms of how the indicators were chosen, the approach taken to present this information in ranked format, and access to the original data are a very crucial point.

Hence, we propose to review and assess existing health system rankings as this may also play a role in improving the transparency and the ability of reproducibility of available health rankings. The objective of this review is to evaluate the transparency of existing health system rankings by assessing the completeness and comprehensiveness of the ranking methodology from a systematic perspective. This may also help giving recommendations for future health rankings, evaluating any current gaps in the literature and to encourage future discussions in this area.

## METHODS

In order to identify existing health rankings a search was performed using different search engines: PubMed, Web of Science, Science Direct, Google scholar and Google. The keywords used to perform the search were the following: “health rankings”, “health system rankings”, “health system performance”, “health system efficiency”. Google and Google Scholar was used to be able to find rankings that were not published in scientific journals. No ranking was found through the other scientific databases (PubMed, Web of Science, Science Direct) in addition to those already found through Google and Google Scholar.

Inclusion was based on the objectives of the rankings. Health systems are not easy to compare, mostly because the health sector produces more than one outcome. The most obvious is the health status of the population, followed usually with some measures of financial protection for the population such as paying out of pocket for care in order to measure the performance of the health system. Based on this definition, only rankings that included a financial dimension to evaluate health system performances as an input-output structure were included. Excluded were rankings that did not focus on health systems or that included only measurements of population health without any financial input.

**Figure 1:**
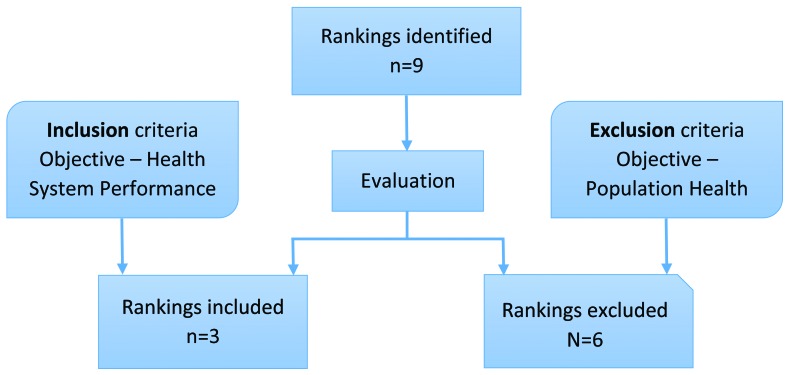
Flowchart indicating health ranking selection process.

A total of nine health rankings were identified when the search was performed. After the evaluation of each ranking, three rankings were selected to be compared, based on the inclusion criteria shown in **Figure 1**.

**Table 1** shows the list of the excluded rankings that had been identified through our key search terms but had mainly a focus on the measurement of population health rather than a focus on the health system performance and did not include any financial dimension.

**Table 1:** List of excluded rankings based on the inclusion and exclusion criteria

Organisation	Title of ranking	Focus (exclusion reason)	Number of countries
Bloomberg	World Healthiest Countries	Measurement of population health / no financial component	145 countries
United Health Foundation	America's Health Ranking	Measurement of population health / no financial component	50 states
24/7 Wall St.	The healthiest (and least healthy) countries in the world	Measurement of population health / no financial component	20 countries
University of Wisconsin Population Health Institute	County Health Rankings	Measurement of population health / no financial component	72 counties
University of Wisconsin Population Health Institute	Wisconsin County Health Ranking	Measurement of population health / no financial component	72 counties
Health Consumer Powerhouse	Euro Health Consumer Index	Measurement of “consumer friendliness”	36 countries

To evaluate the transparency of the methodologies, four categories of evaluation were chosen. They were based on information that is necessary to be able to replicate a ranking. The four categories of evaluation are the following: (1) General information, (2) Statistical methods, (3) Data and (4) Indicators. For each category a number of criteria were established. Scores were determined for each criterion on a scale of 0 for No and 1 for Yes, with a higher score representing better attainment in each category. Scores attained by each category were then added and divided by the number of criteria taken into account as some criterion were not applicable (N/A) to get a comparable average score. The highest summation was considered the most complete and transparent methodology. **Table 2** shows the list and description of all the categories and the criteria that compose them.

**Table 2:** List and description of categories used for the comparison of the rankings

Categories
**General information:**
Information available, open and clear for the description of the processes used
**Criteria:**
a) Published scientific literature
i. Methods for statistical analysis performed
ii. Methods used to determine weights assigned to each indicator
b) Applied weights
i. Reported
c) Surveys (questionnaires administered to the general population used to determine indicators used and weight assigned to each indicator)
i. Published and openly accessible
ii. Methodology for the use of survey data published
**Statistical methods:**
Sufficient information to replicate statistical methods used
Criteria:
a) Used statistical software mentioned
b) Used models mentioned
c) Formulas provided
d) Uncertainty intervals calculated and provided
e) Sensitivity to weight change calculated and provided
f) Sensitivity to different statistical methods calculated and provided
**Data:**
Values used to perform the ranking
Criteria:
a) Data taken from official data banks (coming from established organizations that have data collection groups that update their data regularly)
b) Data taken from the same year (if not using time-series data)
c) Data sources provided
d) Raw data available
e) Years of data used provided
**Indicators:**
Measures to evaluate a health system
Criteria:
a) Reason for the use of each indicator provided
b) Results and calculations for each indicator provided
c) Individual ranking for each indicator provided

## RESULTS

**Table 3** shows the general characteristics of the three selected health system rankings.

**Table 3:** General characteristics of the selected health system rankings

Last year of publication	First year	Organization	Title of ranking	Focus	Objective	Countries	Country First Place	Data source	Data	Number of Indicators
2000	2000	WHO	Measuring Overall Health System Performance of 191 countries - Health Systems: Improving Performance	System Performance	Assess performance of Health Systems in 191 countries	191 countries	France	WHO, WHO member state national vital registration system, WHO administered surveys	1993-1997	5
2014	2004	Commonwealth Fund	Mirror, mirror on the wall	System Performance	Compare performance of 11 developed countries	11 countries	UK	CWF surveys, WHO, and OECD	2010-2013	80
2015	2013	Bloomberg	Most efficient health care 2014	System Performance	Rank countries based on the efficiency of their health care systems.	51 countries	Singapore	World Bank, International Monetary Fund, World Health Organization, Hong Kong Department of Health	Not provided	3

The WHO published the year 2000 the “World Health Report – Health Systems: Improving Performance” which had as objective the assessment of the performance of the 191 WHO member countries used overall five indicators [13]. The Commonwealth Fund (CWF) had its first publication of their ranking entitled “Mirror, Mirror on the Wall” in 2004 and has kept on publishing; their last publication was in 2014 [14]. Its aim is to compare the health system of 11 industrialized countries by using overall 80 indicators to make its comparison. The third selected ranking was published by Bloomberg with the title “Most Efficient Health Care 2014” [15]. It compared 51 countries and ranked them according to what they considered to be efficiency of health care system by taking into account only three indicators.

**Table 4** shows the categories and criteria established for the comparison of the three selected rankings. In the *General information* category, we can see that the WHO scored 1 for every criterion, the CWF scored 1 in every criterion with the exception of the open accessibility of the surveys conducted towards the population. The criterion “Methods used to determine weights assigned to each indicator” received a “not applicable (N/A)” as all indicators were weighted equally. The Bloomberg ranking received the least points in this category. For published literature they received 0 and for reporting of weights a 1. Regarding the surveys, they received N/A because they did not conduct any surveys to perform their ranking.

**Table 4:** Categories and criteria established for the comparison of the three selected health system rankings

Categories	Ranking		
**WHO**	**CWF**	**Bloomberg**
**General information**	Score: Yes = 1, No = 0		
**Published literature:**			
Methods for statistical analysis performed	1	1	0
Methods used to determine weights assigned to each indicator	1	N/A	0
**Weights:**			
Reported	1	1	1
**Surveys:**			
Published	1	1	N/A
Openly accessible	1	0	N/A
Methodology for the use of survey data published	1	1	N/A
***Average score: General information***	***1***	***0.8***	***0.3***
**Statistical methods:**			
Statistical software used mentioned	0	0	0
Models used mentioned	1	1	0
Formulas provided	1	0	0
Uncertainty intervals calculated and provided	1	0	0
Sensitivity to weight change said to be calculated	1	N/A	0
Sensitivity to different statistical methods said to be calculated	1	1	0
Sensitivity to different statistical methods results provided	1	0	0
***Average score: Statistical methods***	***0.9***	***0.3***	***0***
**Data:**			
Data taken from reliable data banks	1	1	1
Data taken from the same year (if not using time-series data)	1	0	0
Sources provided	1	1	1
Raw data available	1	1	1
Years of data used provided	1	1	0
***Average score: Data***	***1***	***0.8***	***0.6***
**Indicators:**			
Reason for the use of each indicator provided	1	0	0
Results and calculations for each indicator provided	1	1	0
Ranking for each individual indicator provided	1	1	1
***Average score: Indicators***	***1***	***0.7***	***0.3***

WHO – World Health Organization, CWF – Commonwealth Fund

For the *Statistical methods* category Bloomberg scored 0 in all criteria as no information of the calculation of the ranking was mentioned or manually found. The CWF scored a 2 overall for “models mentioned” and “sensitivity to different statistical methods said to be calculated”, and it received a N/A for “sensitivity to weight change said to be calculated” because they weighted every indicator equally. The WHO received the scored 1 in every criterion except for the criterion “Statistical Software used mentioned.” The ranking from the WHO scored in each criterion a 1. The CWF scored 0 in “Data taken from the same year” and 1 in the others. Bloomberg scored 0 in “Data taken from the same year” and “Years of data used provided”, it scored 1 in the other two remaining criteria.

For the final category *Indicators*, the WHO scored 1 in each criterion. The CWF scored 0 in “Reason for the use of each indicator provided” and 1 in each of the others. The Bloomberg ranking scored 1 in “Ranking for each individual indicator provided” and 0 in the others.

**Table 5** demonstrates the average score for each category and for the overall comparison. From the scores, we can see that the methodology and information provided by the WHO ranking scores highest in comparison with the CWF and Bloomberg. A higher score means criteria were better met (from 0 until 1 being best). Criteria that did not apply to the ranking were marked as N/A.

**Table 5:** Overall average score of the comparison

	Rankings		
**Categories**	**WHO**	**CWF**	**Bloomberg**
General information	1	0.8	0.3
Statistical methods	0.9	0.3	0
Data	1	0.8	0.6
Indicators	1	0.7	0.3
**Overall**	**3.9**	**2.6**	**1.2**

WHO – World Health Organization, CWF – Commonwealth Fund

Overall the WHO methodology scored the highest according to the criteria that were chosen, 3.9 out of 4. It also scored the highest in every category. The CWF was next with an overall mean of 2.6. Bloomberg had the lowest score with 1.2 with no points in the statistical methods category.

## DISCUSSION

In this paper we have established a set of criteria used to compare the transparency of the published methodologies of health system performance rankings. After having set the inclusion and exclusion criteria, we looked at the methodology of the three selected health system rankings. The rankings were the following: “Health Systems: Improving Performance” by the WHO [13]”, “Mirror, Mirror on the wall: How the Performance of the US Health Care System Compares Internationally” by the CWF [14] and “the Most efficient Health Care” by Bloomberg [15]. The choice and number of indicators for each ranking were very different. The WHO used in total 5 indicators plus 2 other variables considered (GDP and education attainment), the CWF used 80 indicators and Bloomberg used 3 indicators.

Our objective was to assess the transparency of the different health system ranking reports regarding the methodology. Transparency is not only about access to the data, it is built on the free flow of enough provided information to understand the process behind a health system ranking. Therefore, it involves detailed general information including definitions, complete access to the methodology and any statistical techniques, the data itself, the ability to search, filter and manipulate the results, and also the explanations for the chosen indicators and the assigned weights.

Therefore, we divided our review criteria in four categories that are important to evaluate the transparency of these rankings. The four categories were (1) General information, (2) Statistical methods, (3) Data and (4) Indicators.

According to our proposed methodology for the comparison of the ranking methodologies we found that the most complete and transparent methodology was that of the WHO. It obtained the highest score in all of the categories. The WHO provided the most complete information compared to the other two rankings. The CWF did average overall, but it lacked mostly on the statistical methodology category. Bloomberg scored poorly in every category, but also, mostly in the statistical method category.

Indeed, the ranking of the WHO seems to be the most complex one. To calculate the efficiency index, the WHO used a fixed effect panel data model in which the health system is seen as a macro-level production unit and in this case, the overall efficiency combines both technical and allocative efficiency [5]. Three variables were considered: outcome indicator, health system inputs, and effect of controllable non health system determinants of health. The variable outcome indicator is represented by the outcome of the health system and was used by calculating a composite index of five indicators which according to the WHO are the 3 main goals of a health system [6]: (1) Health (level and distribution) (2) Responsiveness (level and distribution) and (3) Financial fairness. They used weights that were assigned to each indicator to calculate the composite index based on Internet surveys and expert opinions. For the variable *Health System inputs* that contribute to producing outcomes total health expenditure per capita (public and private) was used. The third variable effect of controllable non-health system determinants of health was measured by considering the educational attainment of the population, which is calculated by the average years in the population older than 15 years of age. The maximum efficiency index, also called frontier of maximum attainment, of the health system was calculated and the best performing country was used as the reference the other countries were compared to it. The frontier of minimum attainment was calculated by assuming absence health system and this is expressed in the calculation of the efficiency score by considering health inequality and responsiveness level as nonexistent. Uncertainty intervals were estimated and to obtain the confidence intervals Monte Carlo simulation techniques were used. In their conclusions the WHO states that health care system efficiency can be increased without increasing health expenditure and that determinants of relative efficiency are what they aim to focus on studying next [5,7].

The CWF ranking used survey data collected from patient and physician and data taken from the WHO and OECD. It assessed 5 dimensions: (1) Quality, (2) Access, (3) Efficiency, (4) Equity and (5) Healthy lives. Each dimension score was calculated by averaging the score of the different indicators used to evaluate health systems. The indicators used were taken from three surveys performed on patients and primary physicians and the Healthy lives dimension was calculated from data obtained from the WHO and OECD. As mentioned earlier, all indicators in this study were weighted equally [14].

The Bloomberg ranking considered three indicators: (1) life expectancy of the population in each country (2) percentage of GDP per capita cost of health care (3) absolute per capita cost of health care. Bloomberg gave each country an efficiency “score,” with a score of 100 representing a perfect system whereas life expectancy accounted for 60%, the second indicator for 30% and absolute per capita cost of health care accounted for 10%. However, the reasons for the choice of indicators and the dedicated weights are not explained making the transparency and reproducibility of this ranking weak in comparison to the previous two described rankings.

In addition, having a high public spending does not mean that countries will have better health [7]. The problem with these rankings is that health expenditure plays a role but it is not the main component of assessing the health outcomes of a population.

This review is not an in depth analysis of the methods used in the three rankings nor it does assess the methodological validity of the statistical methods including indicators and weights used. In addition, the conduct of health system rankings may be culturally restricted. It is to be noted that we may have missed rankings in other languages that are not included in our review as the initial search was done in English. Moreover, we used a very restrictive definition of health system performance, meaning that a ranking should include at least a financial dimension to be included in this review.

The dates of publications of each health ranking are fairly recent and became a source of great debate after the WHO published its first report [16,17]. Indeed, health system rankings may be seen as controversial as not everyone agrees that the performance of a health system can be quantified and compared in an international context. It may be unclear why a particular definition was chosen, how well it is founded, by whom it was decided and how open and reflective the decision process was. In particular, as no gold standard exists for health system rankings, there are several points to be taken into consideration: first, the choice of indicators rests with those doing the ranking. Consequently, the set of indicators used will vary according to the value system of the person or group doing the ranking. Second, the choice of weights is itself a value judgment and thus can vary depending on who is making the decision. Depending on the number of criteria and their weights, one dimension may dominate all the others, or several trivial dimensions may swamp more crucial ones.

However, such rankings may have considerable influence and may be used to capture public attention and to “sell” magazines or capture advertising revenues by attracting “views” on the internet. While the lack of appealing alternatives has legitimated the use of rankings in the eyes of many, there is still a lively debate over the issue of how to rank in the mainstreaming media as well as in academic circles [17-19].

There is no doubt that there is an increased interest in international health system rankings. In particular, in times of globalization such as the use of internet, but also travel and migration, have given the citizens and patients of many countries an image of life in other countries [20]. As stated by Papanicolas and Smith this exposure and trend towards a globalized world has put health systems around the world under pressure to deliver what is available elsewhere.

Despite of some research initiatives such as the “European Community Health Indicators” (ECHI project), the availability of data for such rankings remains a key challenge [21]. Rankings may be based on convenient data or, in the case of international rankings, on data that is available in a wide range of countries. There is a serious problem of available statistical information at international level that is objective, independent and comparable among countries at the same time. Definitional inconsistencies of measurements across countries may also exist [22].

Another challenge in some of these ranking publications is the fact that they do not go through a peer review board like scientific articles published in journals, and this impedes exchange among scientist and constructive criticism with regards to how data are used, indicators are chosen, weights are determined, and what kind of statistical methods are chosen to be used. It should be also noted, that the ranking of the WHO has been only performed once in 2000, also the Bloomberg ranking was not conducted on a regular basis. Perhaps this may underline the critical and sensitive issue of an international health system ranking whereas other internal rankings comparing the population health within one single country have been repeatedly done [23,24].

In the area of academic rankings that compare the quality and performance of universities, the UNESCO initiated an International Ranking Expert Group as there was a growing criticism of the existing approaches to and methodological problems. In 2006 they adopted a document containing principles of quality and good practice called the Berlin principles on ranking of higher education institutions [25]. We strongly recommend for future studies or expert groups to develop such principles and recommendations in the area of health system rankings.

## CONCLUSIONS

To the best of our knowledge, this review is the first that assesses the transparency of existing health system performance ranking methodologies, which is important for the advancement of the health system research field.

Based on this review, an in-depth evaluation of the statistical methods used in each ranking would be insightful to know how accurate the applied statistical methods are in assessing performance of health systems. Also a report on the comparison of how weights are chosen would be valuable.
